# Subarachnoid hemorrhage secondary to Brucella-induced cerebral aneurysm: a case report

**DOI:** 10.1186/s12879-021-06415-x

**Published:** 2021-07-31

**Authors:** Yanyan Guan, Nannan Xu, Yongyuan Yao, Feng Zheng, Fengzhe Chen, Wei Wang, Xiaomeng Dong, Gang Wang

**Affiliations:** 1 Department of Infectious Disease, Rizhao People’s Hospital, Rizhao, 276800 Shandong China; 2 Department of Infectious Disease, Qilu Hospital, Cheeloo College of Medicine, Shandong University, Jinan, 250012 Shandong China; 3 Department of Intensive Care Medicine, Rizhao People’s Hospital, Rizhao, 276800 Shandong China; 4 Department of Hematopathology, The University of Texas MD Anderson Cancer Center, Houston, TX 77030 USA

**Keywords:** *Brucella*, Cerebral mycotic aneurysm, Subarachnoid hemorrhage, Case report

## Abstract

**Background:**

Brucellosis is a common zoonotic disease that is prevalent in many areas worldwide. This infectious disease can occasionally affect the central nervous system but intracranial arteries are rarely involved.

**Case presentation:**

A 17-year-old female who had a history of recurrent fever for 1 month was admitted for subarachnoid hemorrhage due to cerebral aneurysm rupture. Surgery was performed to fix the aneurysm, but the patient had persistent fever after the surgery. Cerebrospinal fluid testing showed a high white blood cell count and elevated protein level but no pathogen was identified in the first two tests. *Brucella melitensis* was identified in the third cerebrospinal fluid culture, and a diagnosis of brucellosis was finally rendered. The patient was subsequently treated with anti-*Brucella* medications and her symptoms improved significantly at the last follow-up.

**Conclusion:**

Although extremely rare, *Brucella*-induced cerebral aneurysms can occur and this should be considered in the differential diagnosis of cerebrovascular accidents, especially in *Brucella* epidemic areas.

## Background

Brucellosis is one of the most prevalent zoonoses and is caused by *Brucella* species infection. *Brucella* has the ability to escape host immune surveillance; thus, *Brucella* can affect any organ system, and infection with *Brucella* tends to be chronic and persistent [1, 2]. The incidence of nervous system involvement is rare in brucellosis, approximately 3–5% [3, 4]. The clinical presentation of neurobrucellosis is diverse and includes meningitis, meningoencephalitis, myelopathy, polyradiculitis, and mononeuritis [5, 6]. Blood vessels in the nervous system can occasionally be involved, and the presentation of vasculitis range from mild inflammation to necrosis with a rare possibility of cerebral aneurysm formation [7].

The diagnosis of neurobrucellosis is based on microbiological evidence of cerebrospinal fluid (CSF), including the isolation of pathogens and the detection of specific antibodies. Although positive culture of *Brucella* is the gold standard for the diagnosis of brucellosis, according to previous studies [6, 8], only approximately 15% of brucellosis cases are diagnosed through the identification of *Brucella* in CSF culture.

Aneurysm formation as a manifestation of neurobrucellosis is rare and subarachnoid hemorrhage caused by *Brucella*-related aneurysm rupture is even rarer [8]. Here, we report, to our knowledge, the first case of CSF-culture-confirmed *Brucella*-induced cerebral aneurysm in a patient, who developed subarachnoid hemorrhage.

## Case presentation

**Fig. 1:**
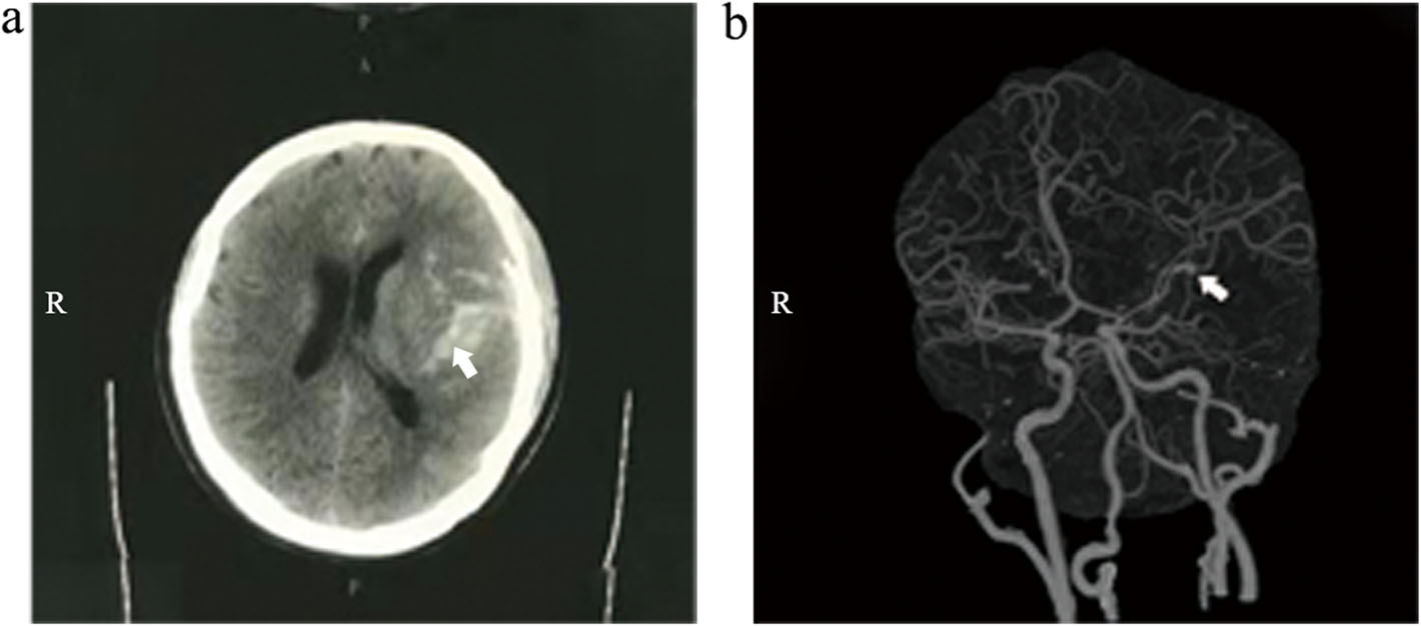
CT and angiography of the patient’s brain. An axial CT scan of the head showed signs of subarachnoid hemorrhage in left fissure and cistern (panel **a**, white arrow). CT angiography illustrated an aneurysm in M2 segment of the left middle cerebral artery (panel **b**, white arrow)

A 17-year-old girl passed out suddenly and was admitted to the emergency room of a local hospital. She presented with persistent fever for 1 month before admission, with headache, fatigue, arthralgia, myalgia and sweating. The overall past medical history was negative, with no family history of hypertension or cardiovascular diseases. An acute computed tomography (CT) scan showed subarachnoid hemorrhage in the left fissure and cistern (Fig. 1).CT angiography demonstrated an aneurysm in the M2 segment of the left middle cerebral artery (Fig. 1). Emergency surgery was performed and the aneurysm rupture was fixed by clipping. After the surgery, the patient had persistent fever. Intravenous cefuroxime (3.0 g q12h) was empirically administered for 2 weeks, but the patient’s condition did not improve. The patient was transferred to our department for further management.

Neurological examination was notable for positive right pathological reflexes, including Babinski’s sign, Chaddock’s sign, Hoffmann’s sign and Kerning’s sign. In addition, the patient had aphasia. Her parents denied a history of infectious diseases or contact with livestock. Laboratory studies showed an elevated erythrocyte sedimentation rate (ESR), at 64 mm/h (normal range 0–20), and mildly elevated liver enzymes, whereas other blood testing parameters, including complete blood counts, C-reactive protein, procalcitonin, creatine, antinuclear antibodies, etc., were within normal ranges. Blood bacterial culture was negative. Abdominal CT scan revealed mild splenomegaly.

Given the negative blood culture and potential infectious aetiology, we performed lumber puncture (LP) to obtain CSF for further examination. Her intracranial pressure was elevated at 320 mmH_2_O (normal range 80–180 mmH_2_O). The appearance of CSF was cloudy with 118 white blood cells/mm^3^ (lymphocyte predominance), protein1.37 g/L, chloridion123mmol/L, and glucose 2.07 mmol/L. Detailed results of the CSF test are shown in Table 1. An advanced diagnostic workup, including CSF cultures for bacteria, fungi and tuberculosis (TB) and polymerase chain reaction for virus, bacteria and TB,was performed without any positive findings (see Table 2). Based on the CSF results, bacterial infection of central nervous system (CNS) was considered and intravenous ceftriaxone (2.0 g qd) was prescribed empirically.

**Table 1:** Cerebrospinal fluid analysis

	Number of lumbar puncture					
	Reference Range	First	Second	Third	Fourth	Fifth
**Color**	Colorless	Cloudy	Colorless	Slightly yellow	Colorless	Colorless
**Turbidity**	Clear	Slight	Clear	Slight	Clear	Clear
**White-cell count (per mm**^**3**^**)**		118	122	80	100	72
**Neutrophils (%)**		8	8	8		
**Lymphocytes (%)**		78	88	78	86	94
**Monocytes (%)**		14	4	14	14	6
**Protein(g/l)**	0.15–0.45	1.37	1.31	1.56	1.34	1.38
**Glucose (mmol/L)**	2.5–4.5	2.07	1.8	1.23	2.02	1.23
**Chlorine (mmol/L)**	120–130	123	121	120	123	121
**Peripheral blood glucose**^**a**^ **(mmol/L)**		5.4	4.6	5.3	5.6	4.8

^a^Peripheral blood sugar corresponding to the glucose level in cerebrospinal fluid

**Table 2:** Results of microbiologic and serologic testing

Variable	Result
**Cerebrospinal fluid**	
**Cultures**	
Bacterium and Fungus	Brucella^b^
**Molecular tests**	
Haemophilus influenzae	Negative
Enterovirus	Negative
Tuberculous bacillus	Negative
Neisseria meningitidis	Negative
Listeria monocytogenes	Negative
Streptococcus pneumoniae	Negative
Varicella-zoster virus	Negative
Mumps virus	Negative
Cytomegalovirus	Negative
Mycoplasma pneumoniae	Negative
Herpes simplex virus types 1 and 2	Negative
Epstein-Bar virus	Negative
JC virus	Negative
Streptococcus agalactiae	Negative
Acinetobacter baumannii	Negative
Cryptococcus neoformans	Negative
Human herpes virus type 6	Negative
Escherichia coli K1	Negative
**Immunological Test**	
Brucella antibody	Positive^b^
**Blood**	
**Cultures**	
Bacterium and Fungus	Negative
**Immunological Test**	
Brucella antibody	Positive

^b^ Cerebrospinal fluid specimens from the third lumbar puncture

After 10 days of treatment, fever resolved and the clinical status of the patient improved. She could communicate with simple words, and her neurological status improved slightly. A second LP with CSF analysis was performed, and the results of the CSF test were similar to those of the previous ones (see Table 1), with a negative result for bacterial culture. Autoimmune-encephalitis-specific antibodies, including anti-glutamate receptors, anti-γ-aminobutyric acid B receptor, leucine-rich glioma inactived-1 and contacin-associated protein-2, were all negative. A re-examination of blood revealed a decreased ESR, at 10.0 mm/h, and other laboratory studies (routine blood parameters, liver-enzymes, procalcitonin, etc.) were negative. The patient was referred to the rehabilitation department for the start of her rehabilitation.

Twenty-five days after the second LP, the patient’s symptoms worsened. She developed fever again, and her body temperature fluctuated from 37.3 °C to 38.2 °C, accompanied by nausea and vomiting. Neurological examination found neck stiffness. A complete blood count showed a total white blood cell count of 3.75 × 10^9^/L, with 34% neutrophils and 60.3% lymphocytes. Magnetic resonance imaging of the brain showed multiple infarction lesions accompanied by regional cerebral cortex necrosis on the left side of the brain. A third LP with CSF analysis was performed, and CSF culture was repeated. This time, the organism *Brucella melitensis* was identified. Given the positive culture, *Brucella* antibodies in serum and CSF were analysed by an enzyme-linked immunosorbent assay (ELISA) kit (IBL International GmbH, Germany). A concentration of ≥12 U/ml was considered positive. The results were positive both in the serum (IgM 10.88 U/ml; IgG>150 U/ml) and in the CSF (IgM 6.37 U/ml; IgG>150 U/ml). Echocardiography showed no sign of endocarditis.

With the confirmed diagnosis of *Brucella*-related CNS infection, triple-agent therapy composed of intravenous doxycycline (0.1 g bid) and ceftriaxone (2.0 g qd) plus oral rifampin (0.6 g qd) was administered. After 2 weeks of treatment, the patient’s body temperature returned to normal. Other symptoms, such as nausea and vomiting were also relieved. A fourth LP was performed and showed that CSF *Brucella*-antibodies were IgM 4.37 U/ml and IgG>150 U/ml. CSF culture showed no growth, as expected. The triple-agent regimen continued for 1 month, followed by the fifth LP, which showed that the level of CSF *Brucella* antibody IgM dropped to 1.49 U/ml and IgG remained >150 U/ml. The patient had no fever, and all symptoms continued to improve. She was discharged and continued on treatment with oral doxycycline (0.1 g bid) and rifampin (0.6 g qd). At the last follow-up, 6 months after anti-*Brucella* therapy, the patient’s condition had markedly improved and her speech recovered.

## Discussion and conclusions

Neurobrucellosis is a rare complication of brucellosis, appearing in only 0.8% of cases of brucellosis in children [9]. Neurobrucellosis usually manifests as meningitis or meningoencephalitis. Cerebrovascular complications are rare, accounting for approximately 3% of neurobrucellosis [10, 11]. Diagnosis may be secondary to acute cerebrovascular events and therefore delayed, resulting in permanent sequelae and even death [6, 12].

However, the diagnosis of neurobrucellosis can be challenging. Neurobrucellosis has neither typical clinical manifestations nor special manifestations in CSF. Its discovery of neurobrucellosis is based on the existence of nervous system manifestations not explained by any other neurological disease, which may lead to a delay in the diagnosis of neurobrucellosis [13, 14]. In the case presented here, the patient had cerebrovascular accidents as the primary symptom and did not have a high risk factor for brucellosis; therefore, brucelosis was not considered in the initial diagnosis. Second, repeated administration of empirical antibiotics led the initial two sets of blood and CSF cultures to be negative. Given the absence of symptom improvement following empirical therapy, antimicrobial agents were discontinued, which may have been the reason for the detection of *Brucella* in the third CSF culture and the subsequent diagnosis of neurobrucellosis. A good clinical outcome of the appropriate medical treatment further supports the diagnosis.

Intracranial aneurysm formation and subarachnoid hemorrhage associated with brucellosis are very rare. According to our literature review, there are only 2 reported cases of subarachnoid hemorrhage due to *Brucella*-related aneurysm rupture [15, 16]. The underlying mechanisms of *Brucella*-related cerebral aneurysm formation are unclear and may be multifactorial. The inflammation reaction can be induced by the bacteria themselves or by the endotoxins they produce and the cytokines they trigger. The inflammation subsequently cause the damage to the muscularis and adventitia of cerebral arteries. The elasticity of vessel walls decreases, and the vessels bulge under the pressure of blood flow to form an aneurysm [3, 15, 17, 18]. Compared with aneurysms caused by other factors, infectious aneurysm progressed more rapidly and had a higher risk of rupture [19] . Although there is still controversy about the optimal treatment [20], dual- or triple-agent therapy should be initiated immediately upon diagnosis. Effective early treatment of brucellosis can prevent the development and rupture of aneurysms and improve the patient’s neurological status [15, 16].

Early diagnosis and treatment can reduce the mortality and morbidity of neurobrucellosis [14]. Therefore, neurobrucellosis should remain in the differential diagnosis when patients come from endemic areas and present with nonspec ific neurological symptoms, even without a clear history of infectious exposure. Early diagnosis is highly critical for these patients to avoid permanent lethality [14, 21]. Given the difficulty of *Brucella* cultivation, antibody detection is of great help especially in CSF [22] .

## Data Availability

Not applicable**.**

## Abbreviations

CSF: Cerebrospinal fluid
CT: Computed tomography
ESR: Erythrocyte sedimentation rate
LP: Lumber puncture
TB: Tuberculosis
CNS: Central nervous system
